# Visual BOLD Response in Late Blind Subjects with Argus II Retinal Prosthesis

**DOI:** 10.1371/journal.pbio.1002569

**Published:** 2016-10-25

**Authors:** E. Castaldi, G. M. Cicchini, L. Cinelli, L. Biagi, S. Rizzo, M. C. Morrone

**Affiliations:** 1 Department of Translational Research on New Technologies in Medicine and Surgery, University of Pisa, Pisa, Italy; 2 CNR Neuroscience Institute, Pisa, Italy; 3 Azienda Ospedaliero-Universitaria Careggi, SOD Oculistica, Florence, Italy; 4 Stella Maris Scientific Institute, Pisa, Italy; McGill University, CANADA

## Abstract

Retinal prosthesis technologies require that the visual system downstream of the retinal circuitry be capable of transmitting and elaborating visual signals. We studied the capability of plastic remodeling in late blind subjects implanted with the Argus II Retinal Prosthesis with psychophysics and functional MRI (fMRI). After surgery, six out of seven retinitis pigmentosa (RP) blind subjects were able to detect high-contrast stimuli using the prosthetic implant. However, direction discrimination to contrast modulated stimuli remained at chance level in all of them. No subject showed any improvement of contrast sensitivity in either eye when not using the Argus II. Before the implant, the Blood Oxygenation Level Dependent (BOLD) activity in V1 and the lateral geniculate nucleus (LGN) was very weak or absent. Surprisingly, after prolonged use of Argus II, BOLD responses to visual input were enhanced. This is, to our knowledge, the first study tracking the neural changes of visual areas in patients after retinal implant, revealing a capacity to respond to restored visual input even after years of deprivation.

## Abstract 2

Implantation of retinal prostheses into patients with retinitis pigmentosa boosts fMRI responses in the thalamus and primary cortex, suggesting that the adult visual brain retains some degree of plasticity after years of deprivation.

## Author Summary

The majority of pathologies leading to blindness are related to diseases of the photoreceptors, such as retinitis pigmentosa (RP). A wide variety of different methods are being developed in the attempt to partially restore vision in blind people, including a retinal prosthesis that uses electrical stimulation of the retina to elicit neural responses in the visual pathway. However, restoring appropriate function to the retina does not necessarily imply that the patients can see again, given that plasticity of the primary visual cortex retained by the adult brain is limited, especially after many years of blindness. Here, we followed the sight recovery process in adult patients with the retinal implant Argus II using changes in blood oxygen levels as a readout of neural activity. The recovery of vision depended on the amount of time and practice the subject experienced with the implant, implying that the reorganization process takes time to develop. Importantly, we observed that subjects who used the prosthetic implant the most were also the ones whose response along thalamic visual pathways and the primary cortex increased before and after sight restoration. Overall, these results suggest that there is residual plasticity in adult subjects and that the adult brain can learn to “see” using an artificial visual input.

## Introduction

A wide range of approaches towards sight restoration are currently being developed, opening new possibilities for blind people with retinal pathologies. These possibilities range from optogenetics techniques [1] through gene therapy [2] to cortical and retinal prosthesis [3,4]. The first retinal prosthesis to enter clinical trials was a 5000-electrode microphotodiode chip (Artificial Silicon Retina [ASR], subretinal approach; Optobionics, Glen Ellyn, Ill, 2000, see [5–7]), and it produced successful results. At present, two devices have been approved as commercial products with clinical trials: the subretinal visual implants Alpha IMS (Retina Implant AG, Reutlingen, Germany, see [8,9]), which induce excitation of the internal plexiform layer, and the Argus II implants (Second Sight, Sylmar, CA, see [10–12]), which induce excitation of the ganglion cell axons with an epiretinal implant, requiring simpler and less invasive surgery.

Most of the retinal pathologies develop late in life, and little is known about the capacity of the adult visual system to process restored visual input after many years of deprivation. Fine et al. [13] described the patient MM, who had his vision restored 40 years after becoming blind at the age of three, when the critical period of vision in human is still open [14]. The study shows that only very limited plasticity was preserved, especially in V1, and that was strictly dependent on the short visual experience during childhood. Cross-modal plasticity [15–18], known to occur also in late blind subjects [19], may raise an additional obstacle to reactivate cortical responses to the restored input [20,21]. The success of a cochlear implant in restoring auditory function correlates well with the level of inactivity of acoustic primary cortices, as assessed by positron emission tomography study [22]. Similarly, the degree of vision loss in retinitis pigmentosa (RP) patients correlates with primary visual cortical responses to tactile stimuli [23,24]. Interestingly, in one case of restored vision (Boston Keratoprostesis, see [25]), responses to motion stimuli were enhanced in extra-striate occipital areas seven months after visual restoration, and this was accompanied by a decreased recruitment of acoustic processing [26]. All these data suggest that, after the original input is restored, the brain needs time to promote a response to the original sensation. This idea is consistent with the results described by Cunningham et al. [23] on two subjects with Argus II Retinal Prosthesis: the first subject (who had the implant only for six weeks before the scanning) showed extensive tactile-evoked responses in V1, whereas in the second subject (who had the implant for 15 weeks), it was largely decreased.

After extensive training, patients with Argus II implants learn to perform a few easy behavioral tasks, such as moving independently in space, locating a large bright square on a screen, and reading large, 100%-contrast characters [10,27–31]. Interestingly, after this learning, the Goldmann visual field perimetry of the operated eye improved in all subjects tested, also well outside the retinotopic region covered by the implant [27]. In one patient, there was also a substantial improvement of the unoperated eye [32]. These findings are in line with the earlier evidence obtained with the subretinal implant ASR [5–7]. These patients were tested for many years after the operation and showed a great improvement of visual acuity and also of the visual field perimetry, assessed by computerized methods, compared with the unoperated eye. Interestingly, the visual field also improved in regions that were not directly stimulated by the implant. Two effects may mediate the improved visibility revealed by these studies: the learning of the artificial visual signals may reopen central visual plasticity, and this need not be retinotopically specific, or release of a peripheral trophic factor may be induced by the injection of current by the implant when in use, and this may diffuse at the not-activated site of the retina [33]. Indeed, Sabel et al. [34] demonstrated an improvement in static perimetry and in visual acuity associated with alpha-band changes in the electroencephalogram (EEG) after only 40 min of noninvasive alternating current stimulation (ACS) delivered transorbitally.

At present, the origin of the increased visibility after Argus II, ASR, or Alpha IMS implants is not clear. In particular, it is not clear whether it is mediated by local retinal mechanisms or by releasing vetoed mechanisms at a central level, such as the thalamus or cortex. In this experiment, we aim to study whether these changes can be traced by measuring Blood Oxygenation Level Dependent (BOLD) visual cortical responses during the course of training with Argus II Retinal Prosthesis. The results show that the thalamus and the cortical responses are enhanced by the use of the implant, pointing to great neuronal plasticity of the adult blind brain.

## Results

**Fig 1:**
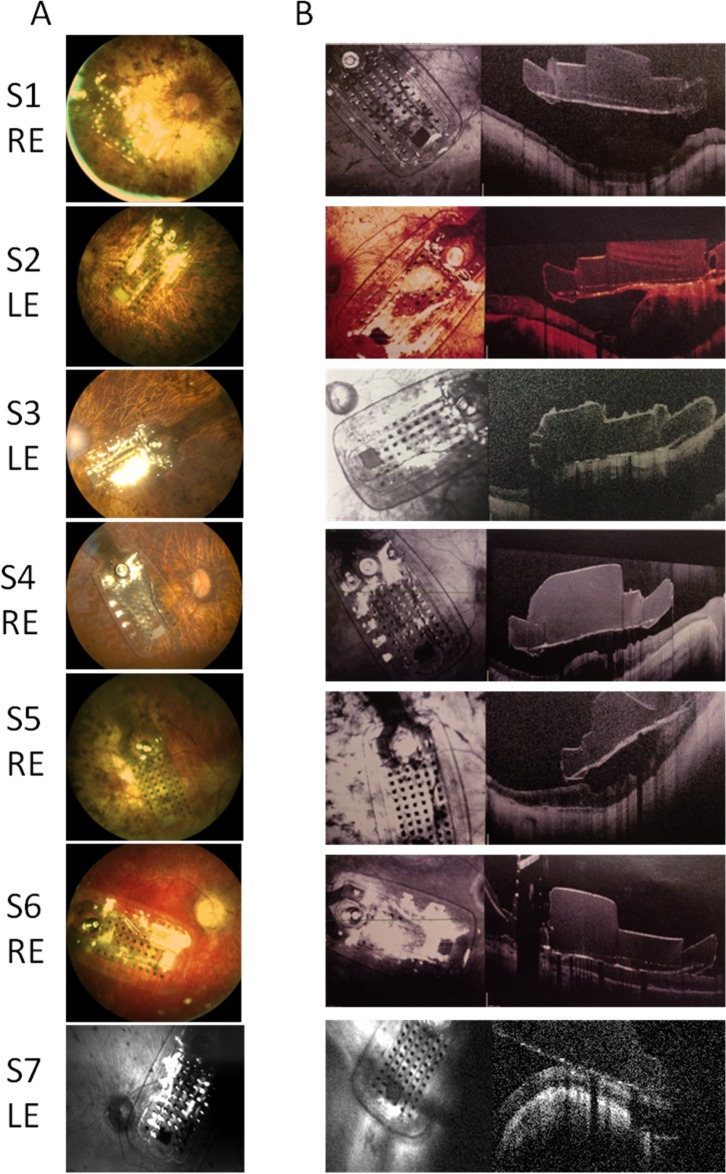
Fundus photographs. (A) Fundus of the seven patients' eyes implanted with Argus II Retinal Prosthesis taken soon after the surgery and (B) at the time of the most recent follow-up. Fundus of S7 is taken at just before the surgical revisions that fixed the adverse event. RE = right eye, LE = left eye.

The surgery was successfully performed in all RP subjects (Fig 1) within similar operating time (see Table 1). No subject had adverse reactions or complications, except for S7, who reported a choroidal detachment two months after the last assessment, which was resolved in two surgery revisions. Six months after surgery, S3 developed a localized retinoschisis behind the implant, which was kept under observation but not treated, because it did not affect the implant effective stimulation. The position of the implant was similar in all patients and presumably excited a similar amount of fibers (see Fig 1).

**Table 1:** Clinical data of patients included in the experiment.

Subject	Age	Gender	Eye	Years of blindness before surgery	Lens status presurgery	Duration of Surgery (Min)	Complications	Treatments and outcome	Number of functioning electrodes
S1	59	M	R	40	Pseudophakic	230	None	None	57
S2	56	F	L	6	Pseudophakic	153	None	None	58
S3	66	M	L	12	Phakic	167	Retinal Schisis	Observation, Stable	56
S4	68	M	R	8	Pseudophakic	113	None	None	58
S5	58	F	R	17	Pseudophakic	110	None	None	58
S6	51	F	R	27	Pseudophakic	144	None	None	58
S7	58	M	L	6	Phakic	110	Choroidal detachment	Two surgery revisions, Resolved	59

**Fig 2:**
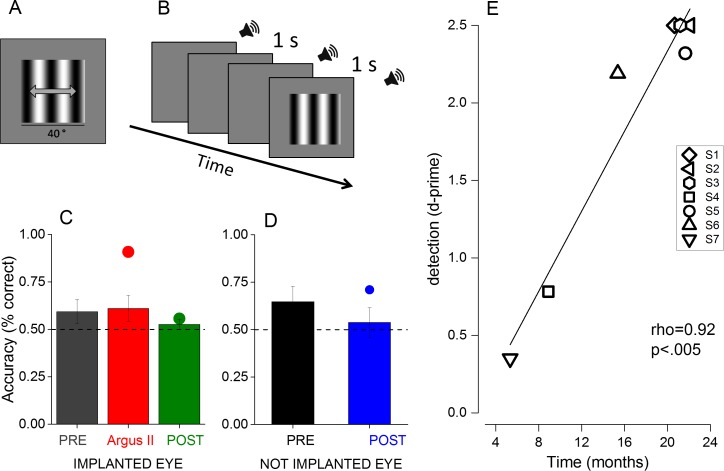
Psychophysical experiment. (A) and (B) Examples of stimuli used in the psychophysical experiment. (A) 2AFC motion direction discrimination task. Subjects had to report the direction of the moving grating (either leftward or rightward). (B) 2AFC detection task. Subjects had to report the interval, limited by sound, that contained the grating stimulus. (C) and (D) Percentage accuracy for the motion direction discrimination task (bar graphs) and for the detection task (dots) for the (C) implanted and (D) not implanted eye. Subjects were tested both before (grey and black) and after (red, green, blue) the surgery both with the Argus II system switched on (red) or off (green). Data represent the average accuracy across subjects (*n* = 7); error bars = SEM. (E) Single subject performance in the detection task expressed in d′ tested with the Argus II system switched on plotted against the time from the surgery.

Given the recruitment criteria of bare light perception (see Methods), before surgery, subjects’ performance for monocular vision was nearly at chance for the motion direction discrimination of contrast modulated gratings (Fig 2C and 2D, gray and black columns). After surgery, no improvement was measured in the motion direction discrimination task either for the implanted eye with the Argus II system switched on or off (Fig 2C, red and green columns) or for the nonimplanted eye (Fig 2D, blue column). The performance for this task was very similar for patients, eyes, and time from the surgery, as shown by the sensitivity measures reported in S1A Fig. Also, contrast sensitivity measured in a detection task in two-interval, two-alternative force choice was nearly at chance level (Fig 2C, green dot) and constant over time (S1B Fig). Consistently, with the lack of cataract, the two phakic patients (S3 and S7) had no change in contrast sensitivity before and after surgery that involved also lens removal (see Methods). All subjects except S7 learned quickly to use the Argus II device, and some simple performance, like the spatial localization of a square of maximum luminance, improved over the testing period (see S2 Fig) when using the implant. Surprisingly, when testing the implanted eye with the Argus II system switched on (Fig 2C, red dot), subjects' performance reached 90% accuracy (t(6) = 6.3; *p* < 0.001) even in more subtle tasks, like the detection of contrast-modulated grating not associated with luminance variation. This improvement in detection performance with the Argus II system switched on correlates significantly (rho = 0.95, *p* < 0.05) with the time from the surgery, suggesting that the effect results from perceptual learning of the artificial incoming signals (Fig 2E).

**Fig 3:**
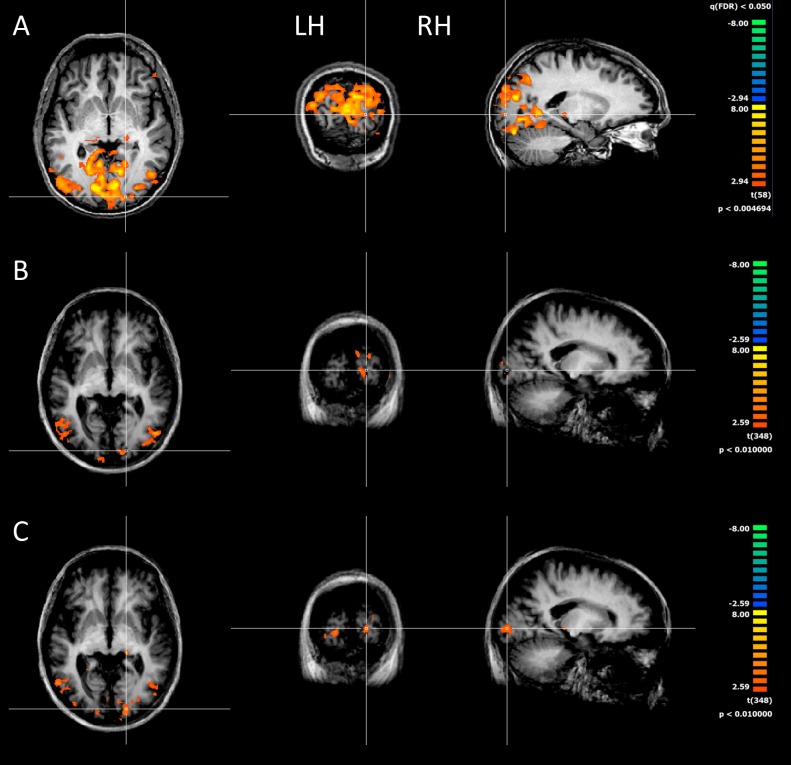
BOLD response to flashing lights. Cortical activity elicited from flashing stimuli in (A) one control subject and (B) in three RP patients before and (C) after the surgery. Although the cortical activity is vastly reduced with respect to the control subject, some responses are detectable in visual cortex of RP patients both before and after surgery.

We recruited four RP patients to measure the BOLD response before and after surgery in response to a sequence of flashes of lights (1 Hz). However, one of these patients did not comply with the procedure of the Argus II training and failed to follow the training routine. For this reason, his data are treated separately and are not included in the functional MRI (fMRI) group analysis. In normal sighted subjects, the flashes of lights massively activate all of the occipital, temporal, and parietal visual brain (*p* < 0.005, corresponding to False Discovery Rate [FDR] q-value of <0.05; Fig 3A). To our surprise, and despite the absence of visual evoked potential (VEP) responses to stronger flashed stimuli than those used in the fMRI experiment, we observed some BOLD responses before surgery in all RP subjects (Fig 3B, average *p* < 0.01, uncorrected). Although the patients never reported seeing the weak flashes (60 cd/m^2^) used to elicit BOLD response, spared activation was observed in the calcarine sulcus, the lateral occipital sulcus, and the mediotemporal sulcus. In all four patients, we performed the fMRI scan again after surgery using the same stimuli with the Argus II system switched off. The activation increased in V1 in the group of three patients who complied with the training. Also, in the LGN, the responses became statistically significant (Fig 3C for the right hemisphere [RH] ipsilateral to the implant, *p* < 0.01, uncorrected).

**Fig 4:**
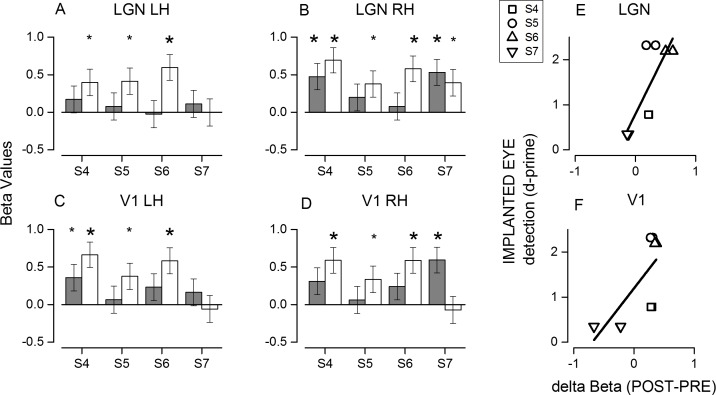
Beta values change after the surgery and correlation with detection accuracy. (A–D) Beta values from anatomically defined ROIs positioned over the left and right lateral geniculate nuclei (A and B) and calcarine sulcus (C and D) before (grey bars) and after (white bars) the surgery for each subject. Asterisks indicate significant modulation by the dark-flash alternation with a *p* < 0.005 (big symbols) or *p* < 0.05 (small symbols). (E and F) Improvement in the detection task performance after the implant with the Argus II system switched on plotted as a function of the increase in beta values in (E) the LGN and (F) calcarine sulcus ROIs. Left and right hemispheres from the same subject are plotted with the same symbols.

To quantify the increase in activation, we measured the average beta values in the anatomically defined regions of interest (ROIs) positioned along the calcarine sulcus and LGN in all four patients. This technique allows detection of changes that may occur at different cortical positions across subjects, as it is to be expected, given the great variability of the perimetry between subjects. The beta values at the level of the LGN (Fig 4A and 4B), both contralateral and ipsilateral to the implant, increased in amplitude after the surgery in the three subjects tested (S4, S5, and S6), reaching statistical significance (*p* < 0.005). A similar increase was measured in the V1 region (Fig 4C and 4D), selected anatomically to encompass the first 20 degrees of visual space representation. Interestingly, a significant response improvement in the LGN and V1 was not observed in subject S7, despite the removal of the lens during the surgery that should have clarity of the ocular media. This subject did not follow the training procedure or use the Argus II daily; he showed no improvement in detection with the Argus II system switched on (triangle in Fig 2E). The result of this subject is clearly at odds with those of the other subjects. The repeated measure ANOVA performed on the data of the three subjects who used the implant shows a significant increase in BOLD activity after surgery (main effect of time, F(1,2) = 26.8, *p* < 0.05; estimated marginal means: before the surgery = 0.18 ± 0.072, after the surgery = 0.516 ± 0.070). We did not observe any significant main effect of the ROIs (F(3,6) = 1.55, *p* = 0.29), indicating that the BOLD increase did not differ between ROIs, nor was it a significant effect of interaction between ROIs and time (F(3,6) = 0.64, *p* = 0.60).

The availability of an RP subject who had a successful surgery but did not use the device provides the rare possibility to disentangle the physical effects of implant and wireless operation from the use of the device as a visual aid in eliciting plasticity. To assess whether the increase in BOLD activity in the LGN and V1 might have a functional relevance, we correlated the BOLD increase with the performance scored in the detection task when testing the implanted eye with the Argus II system switched on (Fig 4E and 4F). The data show that the subjects who performed the detection task with greater sensitivity (with Argus II on) also have higher BOLD responses in the LGN and V1. To assess the significance of this effect, we performed a two-way repeated measure ANOVA on BOLD changes, with the factors visual sensitivity and ROIs (hemisphere being a within factor), and obtained a significant effect for the visual sensitivity factor (F(3.1) = 45, *p* = 0.005). The effect of ROIs and the interaction between ROIs and sensitivity were not significant.

**Fig 5:**
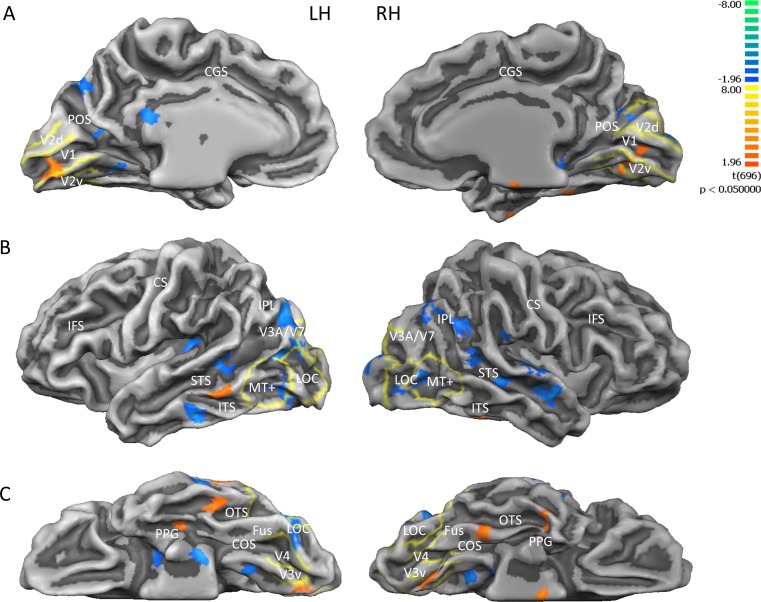
Direct BOLD contrast before versus after the surgery. Results from the multi-study, multi-subjects GLM when contrasting BOLD before versus after the surgery shown on the (A) medial, (B) lateral, and (C) inferior view of a single subject's inflated brain. Map threshold is set at *p* < 0.05, uncorrected. Blue indicates greater BOLD before surgery, red/orange after surgery. Yellow lines identify visual area borders defined by standard retinotopy in normal subjects. Beside the labelled retinotopic regions, activity changes were detected in (A) medial view: retrosplenial cortex, parieto-occipital sulcus; (B) lateral view: inferior temporal sulcus, middle temporal gyrus (red: x = -59; y = -40; Z = -7), superior temporal sulcus, superior temporal gyrus, insula; and (C) inferior view: lateral occipitotemporal gyrus, occipitotemporal sulcus (red LH: x = -45; y = -35; Z = -19; red RH: x = 38; y = -9; Z = -26), fusiform gyrus (red RH: x = 35; y = -35; Z = -19), collateral sulcus. LOC = lateral occipital complex; Fus = Fusiform Gyrus; COS = collateral sulcus; OTS = occipitotemporal sulcus; PPG = parahippocampal gyrus; ITS = inferior temporal sulcus; STS = superior temporal sulcus; IPL = inferior parietal lobe; CS = central sulcus; IFS = inferior frontal sulcus; CGS = cingulate sulcus; POS = parieto-occipital sulcus.

We also performed a direct BOLD contrast between the activation before (blue) and after (red) surgery in the three individual subjects (Fig 5), mapping the results on the average brain where average retinotopy acquired by our laboratory has been projected (see Methods). After surgery, the activity improved in the primary visual cortex and in the mediotemporal and occipitotemporal sulcus. The improvement is statistically significant (*p* < 0.05) but did not survive FDR correction. However, the anatomical localization of the increased activity voxels is not random, but clustered within the primary and secondary visual cortex, including V1, V2, and V3 in the peripheral visual field representation. This corroborates the ROI ANOVA result, suggesting an increase in BOLD activity in these areas after surgery. In addition, given that the retinotopic position of the improvement will vary across subjects, a group analysis is not the optimal technique to detect the possible BOLD changes, and the ROI analysis is more appropriate. Similarly, many higher associative visual areas (like lateral occipital [LO], V3AB, V7, and MT+) showed higher signal before the surgery, suggesting a suppression of these area by the new visual activation (see Discussion).

To rule out the possibility that these results were due to magnetic field interference between the magnetic gradients and the implant (which has local coils and wireless hardware), we performed additional scans, decreasing the number of slices for the subject S4 (S3 Fig) and changing the slice orientation or the visual display for the subject S6 (S4 Fig). No difference was observed in the left and right LGN (t(116) = 0.068, *p* = 0.9; t(116) = 0.20, *p* = 0.8), nor in the left and right calcarine sulcus (t(116) = 0.51, *p* = 0.6; t(116) = 0.49, *p* = 0.6). Similarly, no differences in beta values were observed in S6 (S4 Fig), nor in left and right LGN (t(116) = -0.1, *p* = 0.8; t(116) = -0.6, *p* = 0.5), nor in the left and right calcarine sulcus (t(116) = 1.04, *p* = 0.3; t(116) = 0.6, *p* = 0.5).

## Discussion

Here, we have demonstrated that the adult visual brain retains a degree of plasticity and is able to reorganize its response to process new and abnormal incoming inputs after many years of deprivation in adulthood. The boost in BOLD response takes a long time and intensive training to appear, being stronger in those subjects who used the prosthetic device more intensely and for a longer time. Our data show that the training with Argus II transfers to more subtle improvements than previously observed [30], including the detection of sinusoidal gratings that the patients never saw before and, importantly, that are not associated with overall mean luminance changes. We observed no improvement for the nonimplanted eye and no improvement for motion direction discrimination, even with the Argus II system on. It has been suggested [35] that the perceptual experience produced by the implant might be distorted due to the axonal stimulation of the ganglion cell axons that travel under the implant, introducing motion smear. This may help to explain the lack of improvement for horizontal motion discrimination observed here.

Recent studies have revealed that the adult V1 cortex retains plasticity with deprivation [36,37], even after a brief period of hours of monocular alteration [38]. The BOLD boost that we observed here reinforces this evidence and shows that the plasticity is retained even after years of deprivation. Other sensory modalities usually recruit the occipital cortex after periods of deprivation [15]. This can take place even in normal sighted subjects with training [39] and also in RP patients with loss of vision at adult age [19]. Interestingly, in the RP patients, as in the congenitally blind, the recruitment of the primary cortex is particularly strong with language and memory areas, as demonstrated by the increase in functional connectivity between the occipital cortex, frontal cortex [40,41], and Broca area [41], rather than with other sensory cortex. The need for extensive and prolonged training, suggested by our data, is probably necessary to allow the remodeling of these spurious multisensory activations. A previous study with cochlear implant [22] on prelingually deaf children demonstrated that the degree of cross-modal plasticity (marked by glucose hypometabolism) predicts the auditory temporal cortex’s capability to respond to auditory stimuli of the cochlear implant and the overall success of the functional use of the implant. Prolonged periods of deafness induced stronger cross-modal reorganization of the acoustic cortex and hampered recovery after the cochlear implant. These results are in close agreement with the present results, establishing that the functional response of the appropriate modality response is a good indicator of the use of the implant and of the plastic cortical response. All our patients had only light-dark perception, which may explain the small residual BOLD activation observed in the presurgical scan. Interestingly, none of the four patients reported perceiving the flashing stimulus, probably because of the low intensity of the flashes. However, some activity was clearly elicited, suggesting that some functional connection between retinal input and the cortex is still present in these RP patients despite the lack of subjective report and the lack of VEP responses. This also indicates that the cross-modal plasticity in V1 demonstrated by many studies [15] did not veto the cortex from responding to the flashes. Overall, our data suggest that if a patient had enough visual experience before blindness and has some residual light perception, once new vision information—even artificial and aberrant—is relayed to the brain, the primary visual cortex can reactivate to endorse a plastic cortical response. The more prolonged the exposure to the artificial vision, the stronger the response of V1 to the artificial visual input. Interestingly, two other patients implanted with Argus II [23] have been reported to show a reduction of tactile cross-sensory plasticity in V1 after use of the implant, suggesting that the first step of the plastic response of the primary visual cortex is the weakening of the cross-modal responses and, later on, the restoration of the original visual modality. These two patients did not show any response to visual stimuli, but they were tested only a few months after surgery, reinforcing the present data that a longer time is need to promote plasticity. We observed an increase of BOLD activity in the V1, V2, and V3 corticies, but not in MT+, LO, nor in associative cortices such as intraparietal, superior temporal, and precuneous cortices. This suggests that higher associative areas that have stronger cross-modal plasticity [18,42–46] may have hampered the visual responses. The lack of a global activation of all the visual cortical circuits may also explain the lack of perception in these patients. It would be interesting to follow these patients for a longer time, implementing a more intense training regimen to verify the hypothesis that eventually these associative cortices may also respond to light, and then, perhaps, perception could also be partially rescued.

The spared plasticity of V1 and low tier associative cortices was not expected, given the prevailing evidence of low plasticity to visual stimuli observed in the two subjects who regained vision at adult age [13,26]. However, both of these studies followed the progression of congenitally blind patients or patients who became blind during the critical period. The data on the subject MM [13] are very clear in stating that even after more than ten years of restored vision, the visual response did not change [47]. Interestingly, the only plastic reorganization was observed in area MT+, specialized for motion processing [16,18]. Development of motion-selective mechanisms is nearly complete at three years, the time of the blindness onset of this patient [14,48]. Even more surprisingly, we observed plastic changes even in the thalamus. LGN plasticity, even during development, is usually believed to be very limited [49]. It is surprising that the effect that we observed here for the LGN is nearly as strong as for V1. One possibility it is that it is mediated by an attentional modulation towards a stimulus known to modulate activity in LGN [50]. However, this is unlikely, because no subject reported seeing any stimulus during the scan despite the BOLD activity. The lack of perception was also confirmed by the total absence of any electroretinogram (ERG) or VEP responses. Another possibility it is that the plastic changes originate at the retinal level, mediated by some local and unknown trophic effect.

The first study to use a subretinal implant observed a clear recovery of visual acuity and perimetry in six out of ten patients over a period of seven years from the surgery [5–7]. The recovery was not confined to the stimulated retinal position, being observed very far from it, but it was confined to the same eye. This suggests that it might be mediated by a retinal trophic effect induced by the surgery itself, by the local injection of current, or even by the overall improvement in eye health after surgery. Ciavatta et al. [33] observed a significant elevation in fibroblast growth factor-2 (Fgf2) expression in rats following implantation of an active micro-photodiode array compared with rats with a minimally active array or sham surgery. These authors suggested that subretinal electrical stimulation by the active array induced selective Fgf2 expression, producing a neuroprotective effect on the retina. Also, an expansion of the visual field perimetry [27] has been observed in some Argus II patients, consistent with the diffusion of retinal trophic factors. However, in one patient, there was also a strong improvement of the temporal retina of the fellow eye as well of the operated eye. The improvement in both eyes declined as the patient used the device less. This latter result is hard to explain in terms of release of a retinal trophic factor and also in terms of electrical stimulation, given that the electrical field produced by the wireless coils attached to the Argus II is very low at the distance of the fellow eye. Interestingly, the improvement occurred for homologue regions of the retina, which might suggest that it is the neuronal activity at higher levels in the visual pathways that might exert a trophic function. For example, the influence of the operated eye on the fellow eye may be mediated by an anterograde effect from a central station such as the LGN, where the projection of the fiber from the two eyes is closely interlayered, and some cross-talk of neuronal discharge may take place. The fact that patient S7 of the present study did not show any improvement in BOLD response does not support the view that plasticity arises from the surgery itself or by the overall improvement in eye health after surgery. This patient had the same successful surgery, the same visibility thresholds, and similar years of deprivation to the other patients. However, he was the only one who did not use the Argus II system. The lack of visual responses in this patient provides a strong control against possible artifacts between the device and the magnetic field, which might have influenced the increase of the BOLD response after surgery. Although at this stage it is very difficult to disentangle the origin of the observed recovery after surgery, our data indicate that the neuroplastic response is present at the early stage of processing.

Whatever the mechanisms mediating the plasticity, it is worthwhile to stress a few important implications of our results. They show that the adult brain is able to reorganize itself to adapt to the new incoming visual stimulation even after years of blindness; this takes place only after extensive training and well before proper perception is achieved; the BOLD signal is more sensitive than a perceptual threshold or EEG measure to monitor these changes. The reason for the superiority of BOLD in monitoring these changes might be that signals from multiple sources, both bottom-up and top-down, are integrated within the BOLD response and over long periods. In addition, this also indicates that some activity related to the visual stimulus reaches the cortex in RP patients despite complete blindness. If so, this suggests that, in RP patients, the input signal may be actively suppressed at the cortical level, probably because it is too aberrant and temporally noisy to mediate a perceptual response.

## Methods

### Subjects

All subjects signed the informed consent for prosthesis implant after being informed about the possible outcomes of the surgery. The protocol was approved by the Ethics Committee of Azienda Ospedaliera Pisana (IRB IRB00010229). The trial was and continues to be conducted in accordance with the Declaration of Helsinki and the national regulations for medical device clinical trials in Italy. The clinical trial is posted in a publicly accessible registry approved by the WHO or ICMJE on www.clinicaltrials.gov (trial registration number NCT01490827) and adheres to the TREND guidelines for nonrandomized trials (https://clinicaltrials.gov/ct2/show/NCT01490827?term=Argus&rank=5).

The MRI and the psychophysical protocol of this study were approved by the Ethics Committee of Fondazione Stella Maris (protocol N 11/2012, IRB00003240), which has been superseded by the Regional Pediatric Ethical Board (IRB 00009689). All subjects signed informed consent to participate.

This study was conducted on a group of seven blind patients (males = 4, females = 3, age = 60 ± 6 years) affected with RP with bare Light Perception, before and after (17 ± 7 month interval) implantation with Argus II Retinal Prosthesis. All subjects were required to have some visual memory, no electro-retinographic response, and residual light perception, although bare. Exclusion criteria included the presence of other ocular disease that might interfere with device function or inhibit postoperative device visualization; history of cystic macular edema; pregnancy or desire to become pregnant; deafness; and uncontrolled systemic disease. The initial screening visit included a complete eye examination, retinal fundus photography, fluorescein angiography, optical coherence tomography (OCT), Goldman full-field visual field testing, and ultrasound (A-scan) axial length measurement. Only patients with axial lengths between 20.5 and 26.0 mm were included. All patients had immeasurable monocular logMAR (logarithm of the minimum angle of resolution) visual acuity (worse than 2.9) before surgery. All preoperative tests were performed with both eyes open and, in pseudophakic patients, several years after cataract surgeries. In order to avoid a further intervention of phacoemulsification a few months later, phakic patients underwent clear cornea phacoemulsification and were left aphakic, given that pars plana vitrectomy is a risk factor for cataract progression [51]. The two phakic patients had a normal lens with only an initial sclerosis that did not impair light transmission. No other eye diseases were present. Subject details are listed in Table 1.

The surgery and the rehabilitation were conducted as previously described [27]. Only one eye was implanted (Fig 1). During the use of the Argus II Retinal Prosthesis system, patients need to wear glasses equipped with a central mounted camera. This camera is connected to a video processing unit carried on the patient's body. The video processing unit converts images captured from the camera into electronic signals and sends it to a transmitter coil attached to the glasses. The transmitter coil sends the information wirelessly to a receiver coil that is sutured onto the sclera with a scleral band. A transcleral cable conveys the signals from the receiver coil to a 6 x 10 grid of electrodes array held on the retinal surface (covering about 11 x 17 visual angle degrees) that is abruptly refreshed at a low frequency (below 20 Hz). When activated, each gold electrode emits pulses, which are thought to directly stimulate the retinal ganglion cells or their axons (but see [35]). The patients usually learn to use the device after intensive training consisting in perceiving and localizing crude high-contrast forms and lights on a screen, and following a post-implant visual rehabilitation with the support of low vision therapists.

### ERG, VEP, and Psychophysical Procedure

ERGs were recorded differentially with gold-coated Mylar electrodes positioned in the lower fornix of each eye. The other eye was closed and served as a reference (interocular PERG: [52]). We also recorded VEPs, with Ag-AgCl electrodes placed 2 cm above the inion (active) and at the vertex. The common ground for all recordings was on the forehead. PERG and VEP signals were amplified (PERG 100,000-fold, VEP 50,000-fold), band-pass filtered between 1–100 Hz (6 dB/oct) and averaged on-line by computer over at least 300 periods. The visual stimulus was a strong flash (500 mJ) positioned at about 20 cm distance from the subject eye and delivered at a frequency of 0.5 Hz. We never measured any ERG or VEP reliable responses in any of the RP subjects, consistent with the diagnosis of total blind or bare light perception, both before and after surgery.

The vision of the implanted and the not implanted eye was tested in psychophysical experiments in separate sessions both before and after surgery. When testing with the Argus II system switched on, the subjects' eyes were patched so that only the activity delivered by the implant could be used.

Subjects sat in front of a large screen (86 x 55 degree, luminance 180 cd/m^2^) at a comfortable distance of 57 cm. The stimuli were contrast-modulated gratings, perfectly linearized by gamma correcting the monitor after collecting the photometric values at all luminance levels (from 5 to 350 cd/m^2^) to avoid abrupt increment of luminance that could aid detection in the blind patients given the residual light perception.

Visual functions were evaluated before and after the implant with a motion direction discrimination task and a detection task, with a single or double presentation two-alternative forced choice procedure. The stimuli were optimized to achieve the best performance for the blind patients or for the Argus II device. In the "Motion Direction Discrimination Task" (Fig 2A), subjects were asked to indicate the direction of a drifting grating. A high-contrast (60% Michelson contrast) grating, 40 degrees wide with spatial frequency of 0.0625 cpd, was abruptly presented for 1 or 0.5 s when testing the Argus II system switched on or off, respectively. The grating drifted rightward or leftward at 1 Hz for the Argus II system on or 4 Hz for the Argus II system off. The long presentation time and slow temporal frequency were chosen to be certain to be inside the sampling frequency and the transient delivery of the device. In the "Detection Task" (Fig 2B) the subjects were asked to specify in which of two intervals (marked by tones) was the stationary, low spatial frequency grating presented (for 1 s). The intervals were marked acoustically by tones and separated by more than 5 s. No feedback was given to the subjects.

Each subject performed three sessions of ten trials each per each eye and condition for the motion direction discrimination task and two sessions of ten trials each for the detection task. We allowed all the necessary time for the subject to reach a decision. Usually they took a very long time, in the order of seconds. This meant that the separation between trials was variable, but always longer than 1 s.

For each subject, the average accuracy was calculated and transformed to detectability, d-prime. A one-sample *t* test against 0.5 was performed in each subject to assess the visual performance in the detection task after the surgery.

We also performed the Square Localization test, used previously with Argus II patients [10,27]. On a touch screen display, a white square 7.3 cm wide was displayed on a black background (100% contrast) at random positions. The patient, at 30.5 cm from the screen with free head movements, was asked to localize the center of the square on the screen with a reach-and-touch movement. The task was repeated 40 times, and the average difference between the square center and the patient’s touch, in centimeters, was automatically computed by the testing software.

### MRI

Four subjects (S4, S5, S6, S7) also underwent MRI examination in a 1.5 T GE scanner before and after surgery.

The MRI compatibility and issues of the Argus II system retinal prosthesis have been previously tested [23,53], and the system has been labeled as an MR conditional device (http://www.mrisafety.com/SafetyInfov.asp?SafetyInfoID=313).

Following the MRI recommended procedures, the scanning sessions lasted only 15 min after the surgery. The Argus II system was switched off at least 2 h before the scanning to allow discharge of the wireless coil current and remained off during the exam. Subjects were instructed to notify the MRI operator if pain or unusual sensation of heat was occurring during the scanning, giving particular attention to the orbital region. No subjects reported any uncomfortable feelings of this kind during the examination. The subjects’ eyes were monitored through an infrared camera to assess discomfort symptoms like excessive blinking or squeezing.

MRI data were acquired using a GE 1.5 THD Neuro-optimized System (General Electric Medical Systems) fitted with 40 mT/m high-speed gradients. Each session included a whole brain set of anatomical images with T1-weighted contrast. T1-weighted scans were acquired using a FSPGR sequence with TR = 8.4 ms, TE = 3.9 ms, flip angle = 8°, and 1 mm^3^ isotropic resolution. Echo Planar Imaging Gradient Echo (EPI-GRE) sequences were used for the fMRI data acquisition (TR = 3,000 ms, TE = 35 ms FOV = 192 x 192 mm, flip angle = 90°, matrix size of 64 x 64, and slice thickness = 3 mm). Head movement was minimized by padding and tape.

During fMRI scanning, 15 s of full-field (20° x 30°) flashing stimuli (maximum range 0.1 cd/m^2^ to 60 cd/m^2^ at 1 Hz: 500 ms on and 500 ms off) alternated with 15 s rest periods of dark six times. Stimuli were displayed through liquid crystal goggles equipped with infrared eye-movement camera (VisuaStim XGA Resonance Technology at a resolution of 800 × 600 voxels, subtending 30° × 22.5° at an apparent distance of 1.5 m, with mean luminance of 30 cd/m2).

Subjects were instructed to keep their eyes open, which were monitored and recorded in all subjects. After the MRI scan, we questioned the subjects about their perception during the scan. All of them reported not perceiving the flashes.

Subjects performed one anatomical scan and two functional scans before and after the surgery.

As reported on the MRI-safety website, the implant may create artifacts and MR signal drop. In line with Cunningham et al. [23], we observed local artifacts of the implant during the EPI sequences that were strictly limited to the patient's implanted eye and did not extend to the cortical or subcortical regions. To quantify the local artifact, we calculated the signal-to-noise ratio (SNR) by dividing the mean pixel intensity value in an ROI by the standard deviation of an external ROI of the same extent (in the air region outside the ghosting artifacts). We measured the SNR before (SNRpre) and after (SNRpost) surgery in the three ROIs: one centered on the eye, the second centered on the LGN ipsilateral to the implant, and the last on the ipsilateral primary visual cortex. The SNR of the ROI centered on the implanted eye was significantly different (SNRpre = 17 ± 11; SNRpost = 1.5 ± 0.3) before and after surgery, whereas the one centered on the LGN (SNRpre = 31 ± 11; SNRpost = 32 ± 11) and the one centered in V1 (SNRpre = 100 ± 8; SNRpost = 100 ± 8) were not, confirming that the artifact did not affect the signal at these levels. To rule out further potential confounds due to interaction between the implant and the magnetic field gradients, we ran control scans in two subjects, varying the scanning condition and parameters. In one subject (S4), fMRI was performed by scanning with a different number of slices covering the brain (13 or 30 slices) while keeping the TR constant. If the BOLD signal were driven by the interaction between implant and the changing gradients, we would have expected a different activation between these two conditions. In another subject (S6), fMRI was performed by scanning with axial or oblique slice orientation, hence including or not including the implant in the field of view (FoV). Additionally, to also eliminate possible artifact induced by the goggles, we also delivered the flash via an optic fiber bundle, previously used by [54].

### Data Analysis

Imaging data were analyzed with Brain Voyager Qx (version 2.8 Copyright 2001–2015 Rainer Goebel). Anatomical images were spatially normalized using the Talairach and Tournox atlas [55] to obtain standardized coordinates for the ROI.

Functional data were preprocessed to compensate for systematic slice-dependent differences in acquisition time (using cubic spline), three-dimensional motion correction (using Trilinear/Sync interpolation realigning data to the first volume of the first scan), and temporal filtered (high-pass filter GLM with Fourier basis set, including linear trend, with two cycles). No spatial smoothing was used.

We analyzed the data with a multi-study, multi-subjects fixed effect GLM with one regressor corresponding to the flashing light blocks. The regressor was convolved with the canonical hemodynamic response function (HRF). The BOLD modulation recorded during scans before and after the surgery was contrasted.

Beta values were extracted from two anatomically defined regions of interest. The LGN was localized individually for each single subject with the help of an expert neuro-radiologist, and a 216-mm^3^ ROI was defined around it. The LGN was identified according to anatomical landmarks, as previously described in literature [56–59], and direct measurements in control subjects in response to flashing lights. Anatomically, the LGN was localized on top of the apex of the lateral recess of the ambient cistern in between the optic radiation laterally and the posterior limb of the internal capsule anteromedially. On coronal view, the LGN was visible above the hippocampus. In control subjects, the anatomical localization of the LGN matched exactly with the area activated when subjects were exposed to flashing lights.

The ROI on the calcarine sulcus was defined from the most occipital pole along the sulcus for about 3 cm, representing about 20 degrees of visual space [60,61].

To assess the significance of the ROI analysis, we performed a two-way repeated measure ANOVA, with two factors: "ROIs" of 4 levels (LGN left hemisphere [LH], LGN RH, V1 LH, V1 RH) and "surgery" with 2 levels (before and after).

To identify the visual areas showing activation changes, we performed cortex-based alignment between the RP patients and a control-sighting subject who underwent standard retinotopic mapping. From all the subjects of our database with retinotopic identification of visual area, we chose the one that more closely aligns with the average location of the border of the visual cortical areas across subjects [54,62]. This procedure aligns the brains of a group using the gyral/sulcal folding pattern of the cortex and not the less precise alignment based on the anterior, posterior commissures and the six points defining the limit of the Talairach space. In order to identify the anatomical regions that were falling outside standard retinotopic boundaries, we performed the cortex-based alignment of the RP patients with the Brain Voyager atlas, providing parcellation maps.

## Supporting Information

S1 TREND Checklist Supporting TREND Statement Checklist. Transparent reporting of evaluations with nonrandomized design.
(PDF)

S1 Data Dataset with original data for Fig 2 and Fig 4 in the manuscript and for all the supplementary figures (S1 Fig, S2 Fig, S3 Fig and S4 Fig). (XLSX)

S2 Data This dataset contains the Brain Voyager statistical maps (nifti-1 format) for Fig 3. (7Z)

S1 Fig Visual sensitivity during follow-up. (A) Sensitivity for motion direction discrimination and (B) two interval force choice detection of the individual subjects for the unoperated (closed symbols) and operated eye (open symbols, Argus II system off) as a function of the time after surgery. All patients show equal deficit and no improvement.
(TIF)

S2 Fig The Square Localization task with the Argus II system on. The error (in centimeters) of the pointing of the center of a white square of 7.3 cm on a black screen as a function of the time from the surgery for the individual subjects. The data at zero have been acquired before the operation. Patient S7 did not comply with the training program. All other tested patients show a decrease of the localization error with time.
(TIF)

S3 Fig Control for artifacts. Beta values from anatomically defined ROIs covering the left and right lateral geniculate nuclei (A and B) and calcarine sulcus (C and D) when scanning S4 with 13 (white bars) or with 30 (hatched bars) slices. Beta values do not significantly differ with different number of slices (keeping the same TR) nor in the LGN or in V1.
(TIF)

S4 Fig Control for artifacts. Beta values from anatomically defined ROIs covering the left and right lateral geniculate nuclei (A and B) and calcarine sulcus (C and D) when scanning S6 using an optic fiber bundle and axial slices (white bars) or MRI-compatible goggles and oblique slices (hatched bars). Beta values do not significantly differ depending on the device used to display the images nor on the slices orientation, including or not the retinal prosthesis in the FoV.
(TIF)

## Abbreviations

ACS: alternating current stimulation
ASR: Artificial Silicon Retina
BOLD: Blood Oxygenation Level Dependent
EEG: electroencephalogram
ERG: electroretinogram
Fgf2: fibroblast growth factor-2
fMRI: functional MRI
LGN: lateral geniculate nucleus
LH: left hemisphere
LO: lateral occipital
RH: right hemisphere
ROI: region of interest
RP: retinitis pigmentosa
V1: primary visual cortex
VEP: visual evoked potential
