# Supplementary figures and images for: Visual BOLD Response in Late Blind Subjects with Argus II Retinal Prosthesis
Source: PLoS Biol. 2016 Oct 25;14(10):e1002569. doi: 10.1371/journal.pbio.1002569 (PMC5079588; doi:10.1371/journal.pbio.1002569)

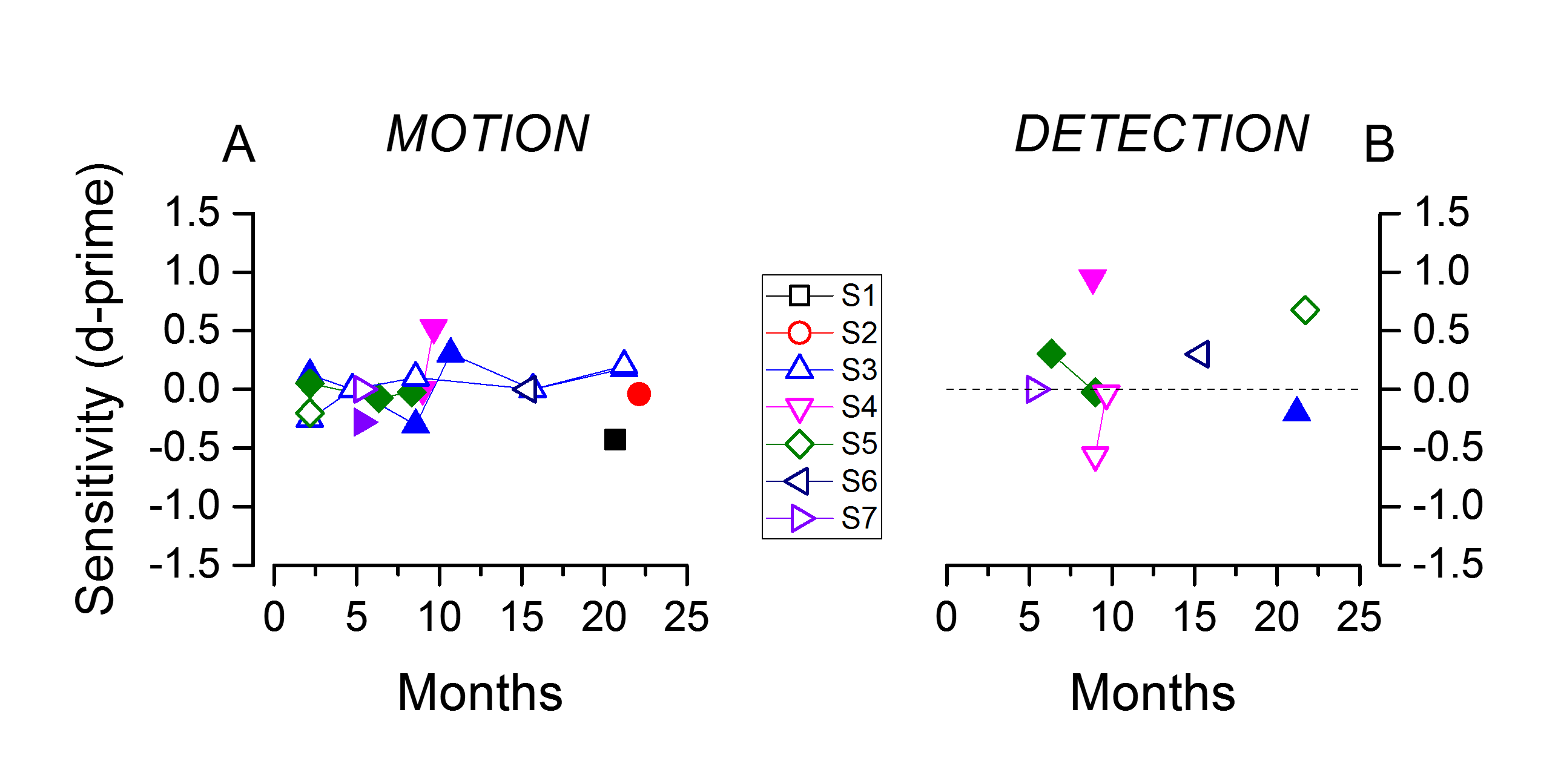

Supplement: S1 Fig — (A) Sensitivity for motion direction discrimination and (B) two interval force choice detection of the individual subjects for the unoperated (closed symbols) and operated eye (open symbols, Argus II system off) as a function of the time after surgery. All patients show equal deficit and no improvement. (TIF) [file pbio.1002569.s004.tif]

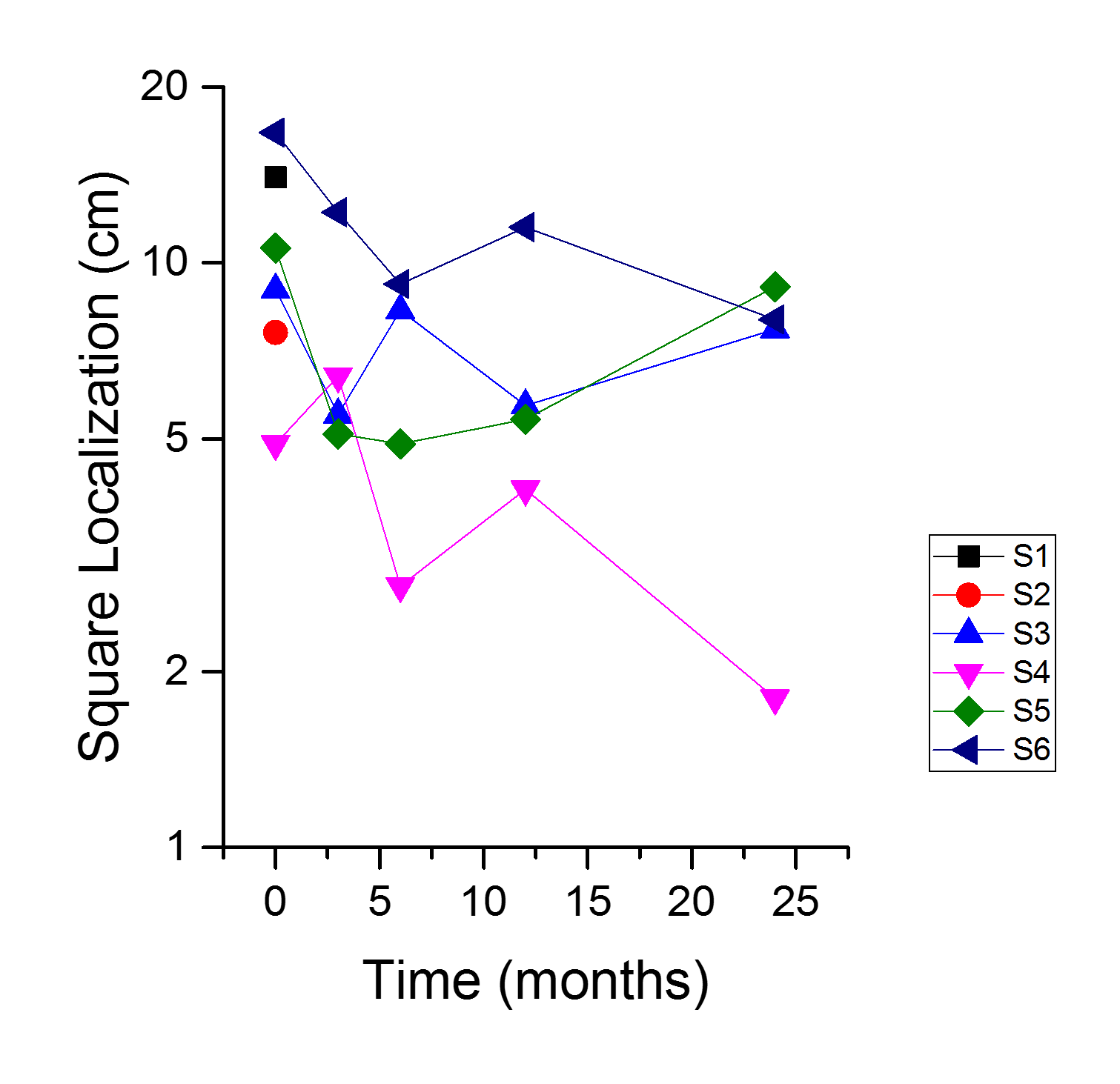

Supplement: S2 Fig — The error (in centimeters) of the pointing of the center of a white square of 7.3 cm on a black screen as a function of the time from the surgery for the individual subjects. The data at zero have been acquired before the operation. Patient S7 did not comply with the training program. All other tested patients show a decrease of the localization error with time. (TIF) [file pbio.1002569.s005.tif]
